# Effectiveness of prescribing physical activity in parks to improve health and wellbeing - the park prescription randomized controlled trial

**DOI:** 10.1186/s12966-020-00941-8

**Published:** 2020-03-17

**Authors:** Falk Müller-Riemenschneider, Nicholas Petrunoff, Jiali Yao, Alwyn Ng, Angelia Sia, Anbumalar Ramiah, Michael Wong, Jane Han, Bee Choo Tai, Léonie Uijtdewilligen

**Affiliations:** 1 Saw Swee Hock School of Public Health, National University of Singapore, Tahir Foundation Building, Block MD1, 12 Science Drive 2, #09-01V, Singapore, 117549 Singapore; 2 Centre for Urban Greenery & Ecology, National Parks Board Singapore, 1E Cluny Rd, Singapore Botanic Gardens, Singapore, 259569 Singapore; 3 Health for Life Centre, Alexandra Health Pte Ltd, 90 Yishun Central, Khoo Teck Puat Hospital, Singapore, 768828 Singapore

**Keywords:** Parks, Urban green space, Public health, Physical activity, Randomized controlled trial

## Abstract

**Background:**

Programs promoting population health through physical activity (PA) and exposure to nature are popular, but few have been evaluated in randomized-controlled trials (RCTs).

**Objective:**

To investigate the effectiveness of a park prescription intervention (PPI) for improving total moderate-to-vigorous PA (MVPA), other PA related behaviors, quality of life (QoL) and cardio-metabolic health among adults.

**Methods:**

Healthy individuals aged 40 to 65 years were recruited through community health screenings and randomly assigned to 1) PPI: face-to-face Park Prescription + invitation to weekly exercise sessions in parks, or 2) control: standard PA materials. After the six-month intervention, participants completed accelerometer assessments, questionnaires on health behaviors and QoL, and health screenings. Independent sample t-tests were used to compare outcomes between groups, with secondary analysis adjusted for co-variates via multiple linear regression. A *p*-value <0.05 was considered statistically significant.

**Results:**

Eighty participants were allocated to each group. Participants with mean age of 51.1 (Standard Deviation: 6.3) years were predominantly female (79%) and of Chinese ethnicity (81%). Participation in the group exercise started at 48% and declined to 24% by week 26. At six-months, 145 (91%) participants attended health screenings for outcome measure collection, and 126 (79%) provided valid accelerometer data. Time spent in MVPA favored the PPI group but this difference was not statistically significant (4.4 (− 43.8, 52.7) minutes/week; when removing 2 extreme outliers 26.8 (− 9.7, 63.4) minutes/week). Time spent in parks (147.5 (2.1, 292.9) minutes/month), PA in parks (192.5 (59.5, 325.5) minutes/month), and recreational PA (48.7 (1.4, 96.0) minutes/week) were significantly greater in the PPI group. PPI also significantly improved psychological QoL (4.0 (0.0, 8.0).

**Discussion:**

PPI improved park use, PA in parks, recreational PA, and psychological QoL but not total MVPA. Future RCTs’ are warranted to investigate PPI in different target populations and to provide further evidence for improvements in health outcomes.

**Trial registration:**

ClinicalTrials.gov NCT02615392, 26 November 2015.

## Introduction

In Asia, non-communicable disease (NCD) prevention is a priority for governments to act upon [1–5] and physical inactivity is a major contributing factor to increases in NCD [6]. About 25% of adults globally and 15% in South-East Asia do not achieve the recommended amounts of physical activity (PA) (at least 150 min of moderate-intensity, or 75 min of vigorous-intensity aerobic PA per week, or an equivalent combination of the two) [7]. Studies among adult Singaporeans also illustrated that about 25% of adults are insufficiently active and that middle-aged people exercise the least [8].

Reviews have consistently identified epidemiological evidence of health benefits associated with exposure to parks and other green space, including reduced negative mental health outcomes, lower levels of overweight/obesity, lower levels of cardiovascular disease, reduced prevalence of type II diabetes and reduced mortality [9–11]. The World Health Organization accordingly recommended a systems approach including a focus on active environments and the creation of high-quality green spaces to promote PA and population health [12]. Despite the accumulating epidemiological evidence and these recent recommendations, the lack of sufficiently high-quality evidence on the association between green spaces and health from prospective studies has been frequently highlighted [13–15]. More importantly, creating green spaces may not be sufficient on its own because many people cannot or do not want to spend time in nature for various reasons [16]. Systematic reviews of interventions aiming to promote the use of parks, green spaces, and PA in urban green spaces have illustrated important gaps in the evidence: a very small number of studies overall, studies that are usually of low methodological quality, and interventions that mainly focus on the creation or modification of green spaces [17, 18]. Thus, it was repeatedly emphasized that understanding the mechanisms of exposure to nature and human health requires robust evidence through rigorous evaluations of interventions [13, 14, 16, 18, 19].

The recently published Global Action Plan on Physical Activity highlights the need for more evidence-based PA programmes in parks [20]. The Park Prescription concept emerged from the collaboration between the U.S. Centers for Disease Control and Prevention and the National Recreation and Parks Association. In 2013 ‘Park Prescriptions’ were defined as ‘Programs designed in collaboration with healthcare providers . . . to utilize parks, trails and open space for improving . . . community health’ [21]. Park prescription programs have become increasingly popular because they promote the use of parks and it is believed that this can improve health through the combined benefits of greater PA while being exposed to or interacting with nature [19]. Park prescription also incorporates the concept of exercise prescription, which has been found to increase PA levels among inactive patients [22–25]. Whilst there have been studies of prescribing PA in parks [26, 27], including one randomized-controlled trial (RCT) among parents of children with a high rate of chronic conditions, to our knowledge no RCT has provided evidence for this kind of intervention in the general population [28, 29].

Singapore has more than 400 parks, which are well maintained and well distributed across the island. Due to its greenery and easy access to parks and green spaces it is also referred to ‘a city in a garden’ [30, 31]. However, research has shown that 63% of Singaporeans visit parks and green spaces only twice per month or even less and few reported to engage in active sports in parks [32]. The Singapore context creates an opportunity to explore the application of the novel and increasingly popular approach of Park Prescription to the general population.

The Park Prescription Trial was conceptualized to address the research evidence gaps and evaluate a carefully developed park prescription intervention (PPI), implemented in the context of community health screenings free to middle-aged adult Singaporeans [32, 33]. The objectives of the trial were a) to determine the effectiveness of PPI on PA related behaviours (including the primary outcome, accelerometer-measured moderate-to-vigorous PA), time spent in parks and PA in parks, as well as mental well-being and physical health outcomes, and b) to understand the mechanisms of impact of the PPI through a comprehensive process evaluation using qualitative and quantitative methods. The current study reports on the effectiveness of the PPI.

## Methods

The Park Prescription Trial follows recommendations for the reporting of RCTs [34] and has been previously described in detail [33]. The following sections provide a brief overview of its methodology.

### Study design and participants

The Park Prescription Trial was a parallel group, two-arm RCT with 1:1 allocation ratio to either intervention or control arm. The RCT was conducted in the community setting in Singapore and participants were recruited following community health screenings free to Singaporean nationals and residents. At the time of the study, health screenings took place across the entire city and participants for the present RCT were recruited from screenings conducted by a large hospital with a catchment population in the northern part of Singapore. Health screenings, together with the recruitment process to our study, had been previously described in detail [32, 33]. Briefly, screening events followed a systematic approach at common community outdoor open spaces or community centers, followed by separate sessions during which residents picked-up their health report. In a first step, participants were approached by the research team based on results from health screening. In a second step, the adapted Physical Activity Readiness Questionnaires (referred to as PAR-Q2) was administered by the research team, which included additional sections to formally assess subjects’ age, whether they were pregnant, whether they had physical disabilities or lower limb disorders and the time they spent exercising on a weekly basis.

After completion of health screening and the PAR-Q2, individuals were enrolled in the Park Prescription Trial if they met the following criteria:

- Singapore citizen or permanent resident;
- aged 40–65 years;
- self-reported weekly exercise < 150 min per week;
- systolic blood pressure ≤ 139 mmHG and diastolic blood pressure ≤ 89 mmHG;
- fasting glucose level ≤ 6.0 mmol/l;
- pass the adapted Physical Activity Readiness Questionnaire (PAR-Q) [35];
- able to write and read in English or Chinese; and,
- provide written informed consent.

After confirming eligibility, participants were randomized (maintaining allocation concealment) into one of the two arms based on computer-generated random sequence using STATA statistical software version 12 [36]. Block sizes were generated randomly using a minimum block size of four and a maximum block size of ten. Participants in both arms completed assessments at baseline (prior to randomization), three-month, mid-intervention follow-up, and upon completion of the intervention at the six-month follow-up. This trial and all its associated forms and resources have been approved by the National Healthcare Group Domain Specific Review Board (DSRB) in Singapore [2015/00611-Park Prescription Trial] and informed consent involved the participants reviewing a participant information sheet, before receiving a brief overview of the study. The trial had been registered with clinicaltrials.gov (NCT02615392).

### Intervention

#### Park prescription intervention group

The PPI was developed based on detailed formative research by Uijtdewilligen and colleagues [32]. Participants in the intervention group received face-to-face counselling on PA. They completed a park prescription sheet with a trained study team member during counseling, where they committed to a goal which specified the frequency, intensity, time and location of exercise in parks. Participants subsequently received information brochures about parks in their neighborhood and a sheet to plan their weekly PA in parks. One brochure that was specifically developed for the trial provided information on the main parks in the northern part of Singapore (within communities where participants were recruited from community screenings) and their features, including walking trails (with time needed to complete them) and locations of fitness corners. The other brochure was a general brochure from the Singapore National Parks Board containing a map and information on the Northern Explorer Loop (a series of parks in Singapore’s north connected by a network of walking and cycling paths). The participants also received a planning sheet, where they filled in the types of activities they aimed to do each week over the trial period. Half-way through the trial a trained study team member provided a brief counselling phone call. The counselling assessed progress towards set goals and included modification of those goals if necessary. In addition, participants were invited to join a weekly one-hour outdoor structured and supervised physical activity program in the park for a period of 6 months. Each one-hour session comprised moderate intensity aerobic activity and strength and balance exercises. The structured PA program took place on one weekday evening and on Sunday mornings in public parks located in the participants’ neighborhood. The sessions utilized different areas and features of the parks, including walking trails and open spaces, to maximize participants’ exposure to greenery. To encourage attendance, participants received Short Message Service (SMS) reminders prior to each weekly exercise session.

#### Control group

Participants in the control group continued with their daily routine. They received standard PA promotion materials, which were existing publications by the Health Promotion Board, Singapore. In addition, they received all the information materials after the PPI group completed the study and they were also invited to join ongoing exercise classes upon study completion.

### Outcomes

Table 1 summarizes the a-priori primary and secondary outcomes, which we previously described in detail by Müller-Riemenschneider and colleagues [33], the instruments used and the measurement time points. The primary outcome for the trial is the mean difference between the PPI group and the control group in time spent in MVPA (minutes per week) objectively quantified via an accelerometer (ActiGraph wGT3X-BT) at six-month follow-up. Secondary outcome measures are defined as the differences between the mean values in the PPI and the control groups at six-month follow-up in health behaviors, mental wellbeing, and physical health.

**Table 1:** Outcome measures at six-month follow-up and their definitions

Outcome	Definition
Primary	
Time spent on MVPA - objective measure	Time spent on MVPA in minutes per week as measured by the accelerometer.
Secondary	
Physical activity related behaviors	
Total volume of PA	Total activity volume as measured by the accelerometer.
Time spent on light and sedentary activity	Time spent per week on light and sedentary PA as measured by the accelerometer.
Time spent on MVPA - subjective measure	Self-reported time (minutes) per week spent on MVPA as recorded in questionnaire.
Time spent in parks	Self-reported time (minutes) in parks in the past month as recorded in the questionnaire.
Time spent being physically active in parks	Self-reported time (minutes) spent engaging in PA in parks in a typical month as recorded in the questionnaire.
Recreational MVPA time	Self-reported time (minutes) per week spent on recreational activity as measured by GPAQ
Sedentary time	Self-reported time (minutes) per week spent sitting as measured by IPAQ
Mental well-being	
Mental well-being	Self-reported mental well-being as measured by SF-12 (1-item, general health), K-10, WHO5 and WHOQOL-BREF
Physical health	
Body Mass Index (BMI)	Weight in kg divided by height squared in m measured by BMI machine.
Fasting blood glucose	Fasting blood glucose in mmol/L. Laboratory assessment
Fasting lipid profiles	Fasting lipids in mmol/L. Laboratory assessment
Systolic and diastolic blood pressure	Systolic and diastolic blood pressure in mmHG measured by a Dinamap blood pressure monitor

Note: *PA* Physical activity, *MVPA* Moderate to vigorous physical activity, *GPAQ* Global Physical Activity Questionnaire, *IPAQ* International Physical Activity Questionnaire, *SF-12* 12-Item Short Form Survey, *K-10* Kessler Psychological Distress Scale, *WHO5* WHO Five Well-Being Index, *WHOQOL-BREF* WHO Quality of Life-BREF

### Data collection and participant follow-up

At baseline, measures of physical health including blood chemistry (blood lipids, fasting blood glucose), blood pressure (systolic and diastolic) and anthropometry (height and weight) were collected prior to enrolment into the trial as part of existing health screenings. Socio-demographic information, data on health behaviors, and wellbeing were collected via self-administered questionnaires after enrolment. The baseline questionnaire combined items from several validated questionnaires. It was piloted with a sample of Singaporeans who were within the same age range as study participants to ensure it was appropriate in the local context and to assess if time to completion was acceptable. All questionnaires used had Chinese translated versions for participants who preferred these.

At the six-month follow-up participants were invited to attend a follow-up health screening. The letter, including the questionnaire, accelerometer and instructions for wearing it were in most cases hand delivered to participants at their homes. During the scheduled health screening visits, participants answered the self-administered questionnaire (if it had not been completed in advance), returned the accelerometer and underwent a health screening.

### Accelerometer procedures and data processing

An explanation on wearing the accelerometer was provided verbally and in writing. The accelerometer was worn on the participant’s non-dominant wrist for seven consecutive days. To increase the chances of collecting complete valid days for each participant, a trained study team member collected the accelerometer and downloaded the data to a laptop with the full ActiLife software installed to check the number of complete days of data provided. If there were less than 4 days, the participant was asked to wear another accelerometer for another seven consecutive days. Data were collected at a rate of 80 Hz and downloaded in raw format (ActiLife version 6.13). Raw data were processed in R using the GGIR package (version 1.6–0) [37]. Raw tri-axial accelerometer signals were auto-calibrated and converted into gravity-corrected vector magnitude units, termed the Euclidean Norm Minus One (ENMO) [38]. Accelerometer wear time inclusion criteria were a minimum of 16 h/day for at least 3 days. Non-wear time was estimated based on the standard deviation and value range of each accelerometer axis, using a 60-min window with 15-min increments. For each 15-min period detected as non-wear time over the valid wearing days, the invalid data were imputed using the mean value of valid data at same time points on other days [37]. We used acceleration intensity thresholds (mg) to classify activity during waking time into SB (ENMO≤25.0 mg), light PA (LPA, ENMO 25.0–100.0 mg) and MVPA (ENMO> 100.0 mg).

### Sample size calculation and statistical analysis

The sample size was estimated based on the primary endpoint of MVPA time per week. After the program, a mean difference in MVPA of 30 (SD: 60) minutes per week between the PPI and the control groups was expected based on existing evidence from PA intervention studies assuming a two-sided alpha = 0.05 and 80% power [39]. To detect this effect, a sample of *n* = 64 per group was estimated and assuming a drop-out rate of 20%, this yielded a sample size of *n* = 80 per group and *n* = 160 participants in total.

Descriptive statistics are presented for demographic variables of the PPI and control groups at baseline using mean and standard deviation for variables that are normally distributed, median and interquartile range for skewed continuous variables, and frequency and proportions for categorical variables. For the continuous primary outcome of MVPA as well as continuous secondary outcomes of health behavior, mental health and physical health measures at the six-month follow-up, the mean difference between PPI and control groups were first evaluated using independent sample t-tests. Further adjustment for respective baseline values of the outcome variables (where available) were made via multiple linear regression analysis.

Pre-specified subgroup analysis based on self-reported baseline engagement in PA in parks was performed to investigate the effect of the PPI on the primary and secondary outcomes. A multiple linear regression model was used, with the interaction between PPI and baseline engagement in PA in parks adjusted.

Since there were two extreme outliers in primary outcome MVPA, with values more than six and nine times of the overall average, the analysis of the primary outcome MVPA was repeated with two extreme outliers removed. R version 3.6.0 (Auckland, New Zealand) and STATA version 14 (TX, USA) were used to conduct the analysis. All evaluations were made on the modified intention-to-treat population, where participants are included according to their assigned group if they provided valid outcome data. Two-sided tests at the 5% level of significance were conducted, effect sizes and 95% confidence intervals are reported for the respective outcome measures [40].

## Results

### Participant flow and characteristics

**Fig. 1:**
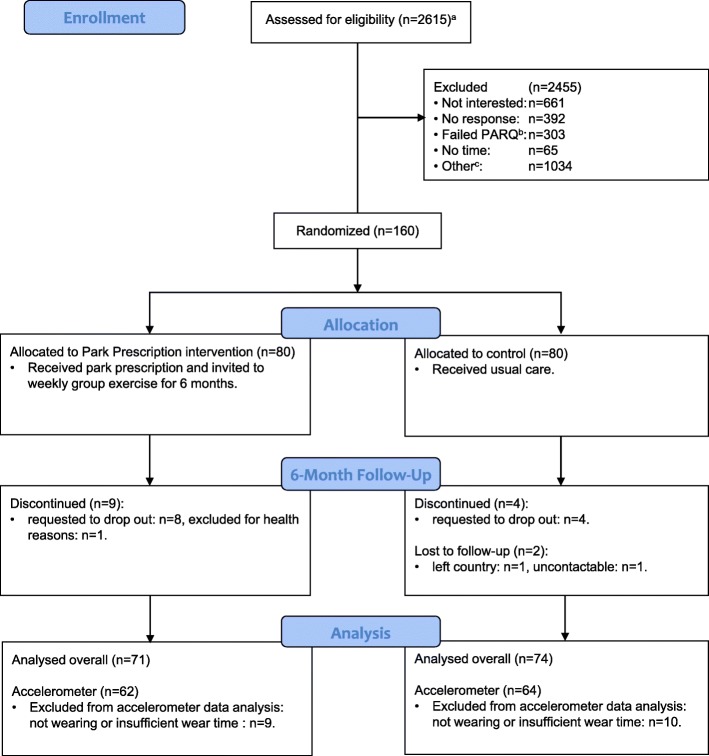
Study flow diagram

Over a period of about 9 months between April and December 2016, approximately 2615 community members participated in targeted health screenings and were approached directly or retrospectively via phone/mail by our team. Among eligible individuals, 160 agreed to participate and were enrolled and randomized to PPI (*N* = 80) or control (N = 80) group. Of those, 145 (PPI:71, control:74) participants attended the six-month follow up. After processing accelerometer data including wear-time validation, 126 (78.8%) participants (PPI: 62, control: 64) provided at least 3 days of valid data and were included in the analysis of the primary outcome (Fig. 1).

Participants mean age was 51.1 (SD: 6.3) years. They were predominantly female (79%), of Chinese ethnicity (81%), married (79%), working (76%), had secondary or lower educational level (52%), and were overweight or obese (56%). The PPI and control group key socio-demographic variables were well-balanced except for Body Mass Index, which was higher in the PPI group. Table 2 presents main behavioral, wellbeing and health related characteristics according to randomization group.

**Table 2:** Baseline participant demographics, behavior, well-being, and health, overall and according to intervention group

Characteristics	Total (*N* = 160)	PPI (*N* = 80)	Control (*N* = 80)
Age	51.1 ± 6.3	52.1 ± 6.5	50.0 ± 6.0
Gender: Female	127 (79)	65 (81)	62 (78)
Race			
Chinese	130 (81)	67 (84)	63 (79)
Malay	14 (9)	7 (9)	7 (9)
Indian	13 (8)	5 (6)	8 (10)
Others	3 (2)	1 (1)	2 (2)
Education			
Secondary and below	84 (52)	41 (51)	43 (54)
Pre-tertiary	46 (29)	25 (31)	21 (26)
University and above	30 (19)	14 (18)	16 (20)
Work status: working	121 (76)	53 (66)	68 (85)
Marriage: Currently married	126 (79)	66 (82)	60 (75)
Household income (in Singapore Dollar per month)			
Below 2000	34 (21)	17 (21)	17 (21)
2000–3999	40 (25)	20 (25)	20 (25)
4000–5999	34 (21)	15 (19)	19 (24)
6000 and above	52 (32)	28 (35)	24 (30)
Physical activity related behaviors			
Total MVPA (min/week)^a^	442.7 ± 534.7	475.7 ± 618.1	409.8 ± 437.2
Recreational MVPA (min/week)^a^	71.3 ± 157.5	85.0 ± 207.1	57.6 ± 81.7
Sedentary time (hour/week)^b^	39.6 ± 22.3	38.7 ± 22.7	40.5 ± 22.0
Time spent in Park (min/month)	171.4 ± 293.8	168.1 ± 303.2	174.7 ± 286.1
PA in Park (min/month)	130.3 ± 261.8	132.7 ± 296.6	127.9 ± 223.6
Mental well-being			
General health from SF-12 (range: 1–5)	2.8 ± 0.8	2.8 ± 0.8	2.8 ± 0.7
K10 total (range: 10–50)	13.1 ± 3.6	12.7 ± 2.9	13.4 ± 4.1
WHO5 total (range: 0–100)	58.3 ± 22.3	58.1 ± 22.1	58.5 ± 22.6
Physical Health			
Fasting blood glucose (mmol/L)	5.05 ± 0.42	5.06 ± 0.41	5.04 ± 0.44
High-density lipoprotein, HDL (mmol/L)	1.56 ± 0.46	1.58 ± 0.50	1.53 ± 0.43
Low-density lipoprotein, LDL (mmol/L)	3.23 ± 0.87	3.44 ± 0.88	3.02 ± 0.81
Triglycerides (mmol/L)	1.27 ± 0.94	1.24 ± 0.60	1.30 ± 1.20
Systolic blood pressure (mmHG)	118.0 ± 11.5	117.8 ± 11.6	118.2 ± 11.6
Diastolic blood pressure (mmHG)	71.3 ± 7.5	71.5 ± 7.7	71.2 ± 7.3
BMI (kg/m2)	23.9 ± 4.1	24.2 ± 4.1	23.6 ± 4.1

Note: Data are mean ± SD or n (%) unless otherwise indicated

^a^Subjective measures based on GPAQ

^b^Subjective measures based on IPAQ

**Fig. 2:**
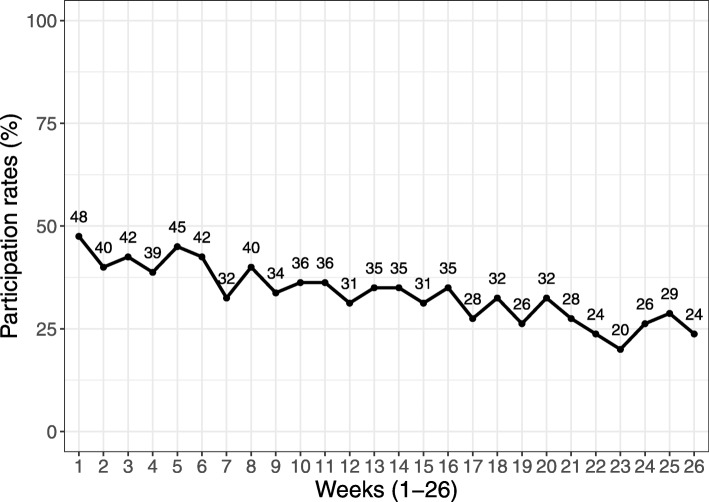
Group exercise participation rates over time (insert around here)

All PPI participants received the face-to-face counselling and park prescription materials following randomization at baseline. Participation in weekly exercise sessions in the parks declined from 48% during the first week to 24% during the last week of the 6-month intervention period (Fig. 2). No cross-over of control group participants to the PPI group was observed during the intervention period.

### Effect of the park prescription intervention on objectively measured physical activity related behaviors

Accelerometer-determined outcome variables are presented in Table 3. On average, the 126 PPI and control group participants with valid data for the primary outcome recorded 7.2 (SD: 0.9) and 6.5 (SD: 1.2) valid wearing days with an average wearing duration of 23.0 (SD: 2.3) and 23.1 (SD: 2.1) hours. Mean minutes of bouted MVPA per week (primary outcome) were greater in the PPI than the control group, but this difference was not statistically significant (4.4 (− 43.8, 52.7) minutes/week, *p* = 0.855 for all participants; when removing 2 extreme outliers 26.8 (− 9.7, 63.4) minutes/week, *p* = 0.148). Accelerometer-determined total volume of PA as reflected by mean ENMO was also higher in the PPI compared to the control group (1.1 (− 2.1, 4.3) mg), but this difference was again not statistically significant (*p* = 0.510). Other accelerometer-determined secondary outcomes including inactivity time and light-intensity PA are presented in Table 3 and did not differ between groups. The results remained consistent in the secondary analyses adjusting for covariates.

**Table 3:** Outcome measures by park prescription intervention (PPI) and control groups at six-month follow-up

Outcome measures	PPI (*N* = 71)	Control (*N* = 74)	Model 1^a^		Model 2^b^	
			Treatment effect	*P*-value	Treatment effect	*P*-value
Primary						
Objective 10 min bouted MVPA (min/week)	114.5 ± 121.3	110.1 ± 150.9	4.4 (− 43.8, 52.7)	0.855	0.6 (−46.5, 47.6)	0.981
Objective 10 min bouted MVPA (min/week)^*^	114.5 ± 121.3	87.7 ± 79.6	26.8 (− 9.7, 63.4)	0.148	25.2 (− 11.4, 61.9)	0.176
Secondary						
Physical activity related behaviors						
Accelerometer valid measure period (day)	7.2 ± 0.9	6.5 ± 1.2	0.7 (0.3, 1.0)	0.001	–	–
Average wear duration (hour/day)	23.0 ± 2.3	23.1 ± 2.1	− 0.1 (− 0.9, 0.7)	0.759	–	–
PA volume, ENMO (mg)	34.6 ± 9.1	33.6 ± 9.0	1.1 (− 2.1, 4.3)	0.510	0.8 (− 2.3, 4.0)	0.595
Inactivity time, 0–25 mg (hour/week)	66.2 ± 13.0	66.7 ± 13.9	− 0.5 (− 5.2, 4.3)	0.845	− 0.3 (− 5.0, 4.5)	0.905
Light PA, 25–100 mg (hour/week)	41.5 ± 8.5	40.3 ± 11.2	1.3 (− 2.2, 4.1)	0.475	1.3 (− 2.2, 4.8)	0.476
Total MVPA (min/week)	814.9 ± 860.8	662.4 ± 775.1	152.5 (− 116.8, 421.8)	0.265	130.2 (− 129.1, 389.5)	0.323
Recreational MVPA (min/week)	142.3 ± 155.4	93.6 ± 131.0	48.7 (1.4, 96.0)	0.044	46.2 (0.3, 92.1)	0.049
Sedentary time (hour/week)	37.7 ± 21.3	44.0 ± 27.1	−6.3 (−14.3, 1.7)	0.122	−6.1 (− 13.5, 1.3)	0.103
Time spent in Park (min/month)	333.9 ± 506.2	186.4 ± 358.4	147.5 (2.1, 292.9)	0.047	156.0 (13.7, 298.3)	0.032
PA in Park (min/month)	333.0 ± 499.3	140.5 ± 270.7	192.5 (59.5, 325.5)	0.005	190.3 (59.7, 320.9)	0.005
Mental well-being						
General health from SF-12 (range: 1–5)	2.8 ± 0.7	2.8 ± 0.9	−0.1 (− 0.3, 0.2)	0.712	− 0.1 (− 0.3, 0.2)	0.631
K10 total (range: 10–50)	14.0 ± 3.9	15.0 ± 4.9	−1.0 (− 2.4, 0.5)	0.199	−0.7 (− 1.9, 0.6)	0.277
WHO5 total (range: 0–100)	56.6 ± 20.1	57.6 ± 20.4	−1.0 (− 7.7, 5.7)	0.769	−1.0 (− 6.9, 4.8)	0.727
QOL_physical (range: 0–100)	71.8 ± 12.3	69.6 ± 11.8	2.1 (− 1.8, 6.1)	0.288	–	–
QOL_psychological (range: 0–100)	65.1 ± 11.8	61.0 ± 12.5	4.0 (0.0, 8.0)	0.047	–	–
QOL_social (range: 0–100)	66.0 ± 13.2	63.1 ± 13.6	3.0 (− 1.5, 7.4)	0.187	–	–
QOL_environment (range: 0–100)	65.9 ± 12.4	64.3 ± 12.9	1.6 (− 2.6, 5.7)	0.449	–	–
Physical Health						
Fasting blood glucose (mmol/L)	4.89 ± 0.52	4.87 ± 0.54	0.02 (− 0.15, 0.20)	0.808	− 0.01 (− 0.17, 0.15)	0.875
High-density lipoprotein, HDL (mmol/L)	1.56 ± 0.53	1.57 ± 0.44	− 0.01 (− 0.17, 0.15)	0.915	−0.01 (− 0.11, 0.08)	0.806
Low-density lipoprotein, LDL (mmol/L)	3.60 ± 0.86	3.31 ± 0.84	0.29 (0.01, 0.57)	0.041	−0.06 (− 0.23, 0.11)	0.459
Triglycerides (mmol/L)	1.15 ± 0.71	1.20 ± 0.72	− 0.04 (− 0.28, 0.19)	0.712	0.01 (− 0.17, 0.18)	0.924
Systolic blood pressure (mmHG)	113.4 ± 12.0	113.1 ± 12.9	0.2 (−3.9, 4.3)	0.912	0.5 (− 2.9, 3.9)	0.770
Diastolic blood pressure (mmHG)	70.0 ± 8.2	71.0 ± 8.6	− 0.9 (− 3.7, 1.8)	0.502	−0.4 (− 2.8, 2.0)	0.727
BMI (kg/m^2^)	24.2 ± 4.2	23.8 ± 4.0	0.4 (− 0.9, 1.8)	0.536	−0.3 (− 0.7, 0.0)	0.074

Note: mean ± SD are presented for each group together with the estimate of the treatment effect and the associated 95% CI

*N* = 126 for accelerometer measures (62 for PPI and 64 for control group)

^a^With two extreme outliers removed in outcome: Objective 10 min bouted MVPA

^a^Model 1 provides the unadjusted estimate using the independent sample t-test

^b^Model 2 adjusts for baseline total physical activity via multiple linear regression

### Effect of the park prescription intervention on self-reported behavioral outcomes

At six-months, 145 participants provided questionnaire-based information on PA, park use, and PA in parks (Table 3). PPI participants reported spending significantly more time in parks than control group participants (147.5 (2.1, 292.9) minutes/month, *p* = 0.047). Similarly, PPI participants reported engaging in significantly more PA in parks (192.5 (59.5, 325.5) minutes/month, *p* = 0.005), as well as overall recreational physical activity (48.7 (1.4, 96.0) minutes/week, *p* = 0.044). A marked difference in self-reported total MVPA favoring the PPI group was also observed but the difference was not statistically significant (152.5 (− 116.8, 421.8) minutes/week, *p* = 0.265). The results remained consistent in the secondary analyses adjusting for covariates.

### Effect of the park prescription intervention on wellbeing and physical health

Wellbeing and physical health outcomes are also presented in Table 3. No difference between PPI and control group was observed with regards to psychological distress and overall QoL. Regarding the domain-specific WHO QoL instrument, PPI participants reported better outcomes on all four domains compared to the control group, but the difference was only statistically significant for psychological QoL (4.0 (0.0, 8.0), *p* = 0.047). With regard to the assessed physical health outcomes a significant difference in LDL-cholesterol favoring the control group was observed, but this difference ceased to exist after adjusting for baseline LDL levels. Furthermore, a borderline significant difference in body-mass index favoring the PPI group was observed in the adjusted analysis. All other results remained consistent in the secondary analyses adjusting for covariates.

### Pre-specified subgroup analysis of the effect of the park prescription intervention

As specified in the protocol [33], subgroup analysis investigated intervention effectiveness among participants who engaged in PA in parks prior to the intervention (PPI: *N* = 28, 35%; control: *N* = 29, 36%) and those who did not engage in PA in parks (PPI: *N* = 52, 65%; control: *N* = 51, 64%). Results in relation to the primary and key secondary outcomes are presented in Table 4.

**Table 4:** Subgroup analysis exploring treatment effects by baseline engagement in physical activity in parks

Outcome measures	Engaged in PA in parks (*N* = 54)^a^		Not engaged in PA in parks (*N* = 91)^b^	
	Treatment effect	*P*-value	Treatment effect	*P*-value
Primary				
Objective 10 min bouted MVPA (min/week)	43.6 (− 38.6, 125.9)	0.298	−15.9 (− 75.1, 43.3)	0.599
Objective 10 min bouted MVPA (min/week)^*^	43.6 (− 18.0, 105.3)	0.165	18.2 (− 26.7, 63.1)	0.427
Secondary				
Physical activity related behaviors				
Accelerometer valid measure period (day)	0.1 (−0.6, 0.7)	0.784	1.0 (0.5, 1.4)	< 0.001
Average wear duration (hour/day)	0.3 (−1.0, 1.6)	0.641	−0.3 (− 1.3, 0.6)	0.479
PA volume, ENMO (mg)	1.8 (−3.6, 7.2)	0.511	0.7 (− 3.2, 4.6)	0.740
Inactivity time, 0–25 mg (hour/week)	−1.5 (−9.5, 6.6)	0.722	0.1 (−5.7, 5.9)	0.982
Light PA, 25–100 mg (hour/week)	0.6 (− 5.4, 6.5)	0.849	1.6 (−2.7, 5.9)	0.464
Total MVPA (min/week)	369.3 (− 67.4, 806.1)	0.097	25.9 (− 310.3, 362.1)	0.880
Recreational MVPA (min/week)^**^	−22.4 (−97.6, 52.8)	0.560	91.4 (33.5, 149.3)	0.002
Sedentary time (hour/week)	−6.3 (− 19.2, 6.6)	0.340	−6.1 (− 16.1, 3.9)	0.230
Time spent in Park (min/month)	66.0 (− 161.7, 293.6)	0.570	198.7 (22.4, 375.0)	0.027
PA in Park (min/month)	74.5 (− 135.1, 284.1)	0.486	265.3 (104.0, 426.7)	0.001
Mental well-being				
General health from SF-12 (range: 1–5)	−0.3 (− 0.8, 0.1)	0.125	0.1 (− 0.2, 0.5)	0.461
K10 total (range: 10–50)	0.1 (−2.3, 2.5)	0.923	−1.6 (−3.5, 0.2)	0.085
WHO5 total (range: 0–100)	− 3.3 (− 14.0, 7.4)	0.547	0.7 (−7.5, 8.8)	0.876
QoL_physical (range: 0–100)	0.3 (−6.1, 6.8)	0.923	3.2 (−1.7, 8.2)	0.203
QoL_psychological (range: 0–100)	3.4 (−3.0, 9.8)	0.295	4.5 (−0.4, 9.4)	0.069
QoL_social (range: 0–100)	−0.0 (−7.1, 7.0)	0.990	4.9 (−0.6, 10.3)	0.079
QoL_environment (range: 0–100)	−2.7 (−9.3, 4.0)	0.435	4.2 (−0.9, 9.3)	0.110

Note: Treatment effect, the associated 95% CI and P-value are from multiple linear regression model, accounting for the interaction between PPI and baseline engagement in PA in parks

^*^With two extreme outliers removed in outcome: Objective 10 min bouted MVPA

^**^Significant interaction between PPI and baseline engagement in PA in parks was found, effect size −113.8 (−209.5, − 18.1) min/week, *p* = 0.020

^a^accelerometer measures: *N* = 43

^b^accelerometer measures: *N* = 83

Effect sizes for accelerometer-measured PA outcomes, i.e. mean ENMO and bouted MVPA, tended to be greater among participants who engaged in park-based PA prior to the intervention as compared to participants who had not engaged in any park-based PA. Especially for the primary outcome of this trial, the mean difference between PPI and control group among participants who engaged in PA in parks was substantial and greater than assumed during sample size calculation (mean difference in MVPA among participants who engaged in park PA at baseline: 43.6 (− 28.1–115.4) minutes/week). However, the difference was not statistically significant.

Effect sizes for self-reported park use, PA in parks, and recreational PA were larger among participants who had not engaged in park PA at baseline and were statistically significant for these participants but not for those who had engaged in park PA at baseline. Similar to measures of accelerometer-determined total PA (mean ENMO and bouted MVPA), total self-reported PA was greater among those who had previously engaged in PA in parks although the difference failed to reach statistical significance.

Regarding psychological distress, wellbeing and QoL, effect sizes of the PPI were consistently greater among participants who had not engaged in PA in parks prior to the intervention. Among participants who had not engaged in park PA prior to the intervention, effect sizes for physical, psychological, social and environmental QoL were also greater. The differences favoring the PPI group reached borderline significance in the case of psychological and social QoL. Considering the absence of any intervention effect in the overall sample upon physical health outcomes, we did not perform additional subgroup analysis for these outcomes.

## Discussion

PA in a natural environment is intuitively good for health and interventions which include a ‘natural exposure’ component have received increasing attention in recent years [19]. The current RCT was designed to strengthen the existing evidence and evaluate the effectiveness of a PPI among inactive but healthy community-based, 40–65-year-old adults on behavioral and health outcomes, including accelerometer-measured PA. In that context, results of PPI on behavioral and health related outcomes were mixed. While PPI resulted in consistent improvements in PA, the effect on the primary outcome of objectively measured total MVPA and on objectively measured total volume of PA (mean ENMO) were smaller than expected and failed to reach statistical significance. On the other hand, PPI resulted in meaningful and statistically significant increases in key secondary outcomes, including recreational PA, time spent in parks, and PA in parks. Additionally, PPI achieved improvements in selected measures of QoL and wellbeing, especially psychological QoL, but it had no effect on cardio-metabolic outcomes.

Considering the increasing interest in park prescription programs in many countries, findings from this RCT provide important new evidence. To our knowledge, the only other RCT of a PPI was conducted by Razani and colleagues [28, 29] among low income families with parents and children who were clinic patients and had high rates of chronic illness. The study reported significant improvements in stress levels among parents after 3 months. However, it did not directly investigate the effectiveness of park prescriptions because both intervention groups received park prescriptions delivered by a pediatrician, a postcard with a map of local parks, a pedometer and a journal. The ‘supported’ group differed from the ‘independent’ group with regard to three outings involving free transportation, phone and text reminders, food, and other activities. Elley and colleagues [39] conducted a high-quality cluster RCT which evaluated a similar concept of PA counselling plus exercise prescriptions without a focus on parks and green spaces among more than 800 middle-aged patients of general practitioners in New Zealand. The authors reported significant albeit slightly lower increases in leisure-time PA when compared to our findings. In a previous systematic review Hunter et al., identified 12 studies evaluating interventions that aimed to increase the use of urban green spaces and PA behavior [17]. The majority of these studies evaluated environmental interventions and the authors identified only one RCT of high-quality. It reported significant increases in the amount of PA of park visitors and the frequency of exercise in parks. These results appear broadly consistent with observed significant increases in park use, PA in parks and recreational PA in our trial, but the earlier RCT did not assess total MVPA via accelerometers or self-report [41]. The authors of the systematic review concluded that interventions aiming to increase the use of urban green spaces may be effective, but they highlighted the need for rigorous evaluations of interventions aiming to promote the use of urban green spaces and PA. They further recommended that such evaluations should, among others, pay attention to appropriate sample size estimations, control group designs, and objective outcome assessments.

The present PPI was carefully designed based on extensive formative research to take population-specific barriers to PA and park use in a busy middle-aged working population into consideration. We also identified comparatively large increases in recreational PA and PA in parks associated with the intervention. Despite the increases in park PA and recreational MVPA, the effect on bouted MVPA (regardless of measured objectively or via self-report) was not statistically significant after 6 months. Several factors could explain this. For instance, the decline in adherence to exercise sessions, which decreased to 24% by the end of the study. It is also possible that the exercise sessions were not of sufficient continuity or intensity to translate into increases in bouted MVPA. Throughout the formative research phase intensity and duration of exercise sessions emerged as a potentially controversial issue. While the goal of the project was to increase bouted MVPA, formative research findings suggested a preference of potential participants for less intensive and short exercise sessions [32]. Although coaches were instructed according to the project goals, this may ultimately also have resulted in insufficiently intense and/or continuous engagement by intervention group participants, especially if they decided not to attend the sessions but instead exercised by themselves, which was highlighted as another preference by a number of participants during formative research [32]. Hence, the intervention may have had a greater effect if it had promoted options for unstructured activity more intensively to better support PPI participants who did not attend exercise sessions regularly. Despite the screening of eligible participants including a criterion that they were physically inactive, we also noted that participants self-reported MVPA at baseline was relatively high. This could have limited the opportunities of PPI to achieve expected increases in objectively measured PA. Similarly, engagement in park PA prior to the start of the intervention and a preference for non-park PA despite enrolling in this study may also have limited the overall effect of our intervention. This was anticipated to some extent and addressed by the results of the pre-specified subgroup analysis according to baseline engagement in PA in parks. The findings of the subgroup analysis were somewhat inconsistent. PPI demonstrated considerably greater effects on accelerometer-measured PA outcomes among individuals who had previously reported to engage in PA in parks, while the effect on self-reported outcomes of recreational PA, park visits, and PA in parks was greater among participants who had not previously engaged in park PA. This could suggest that individuals who already use parks for PA can be encouraged to engage in greater and higher intensity PA (bouted MVPA) but that those who do not usually engage in park PA may only increase their park use and park PA somewhat, but not in ways that would also translate into greater bouted MVPA. Overall, these points of discussion suggest that certain modifications to the developed park prescription program and/or the target population may help to improve its effectiveness. Especially since the participation rates in the group exercise sessions were low, which suggests that other elements of the intervention (all of which had almost 100% participation) may have been a mechanism for these effects. We explore this and other mechanisms of impact for the intervention on its outcomes in a separate process evaluation (not yet published) which may inform such modifications.

In addition to the effects on PA and park use, there is increasing interest in the health benefits of parks and other urban green spaces because epidemiological evidence suggests that exposure to green spaces in urban environments is associated with physical and mental health benefits [9, 42]. However, there remains a lack of methodologically rigorous prospective studies [43]. The Park Prescription Trial investigated distress, wellbeing and physical health outcomes as secondary outcomes to strengthen the existing evidence base. The trial demonstrated improvements in selected wellbeing outcomes, particularly psychological wellbeing. No improvements were observed in relation to psychological distress and cardio-metabolic outcomes. To our knowledge, there is no consensus as to what constitutes a recommended amount or clinically meaningful increase in park use and PA in parks. However, the effect of the PPI on these behaviours was substantial and greater than in most previous studies aiming to promote park use and PA in parks [41, 44, 45]. While our results in relation to health outcomes appear to differ from the only other park prescription intervention study that reported reduced stress levels, this may be a reflection of very different target populations [29]. Recreational PA is also known to be associated with positive cardio-metabolic outcomes [46]. Given the considerable increases in recreational PA and PA in parks, the lack of effects of PPI on other health outcomes apart from psychological QoL may appear surprising. However, considering a healthy population with no existing cardio-metabolic diseases and low distress levels, the duration and possibly the intensity of the PA may simply not have been enough to achieve improvements in these outcomes. In fact, the previously cited cluster-RCT of exercise prescriptions, for instance, made similar observations with no effects on cardio-metabolic and positive effects only on selected QoL outcomes. Similarly, considering that our study was not powered to provide definitive evidence for the effects of PPI on wellbeing further prospective and sufficiently large studies are warranted to confirm our observations.

The Park Prescription Trial has several strengths, including the RCT design, adequate sample size, allocation concealment, objective and valid outcome measures and a sufficiently long follow-up [33]. The trial is also conducted in a community setting and thus more likely to reflect real-life effectiveness than strictly controlled efficacy studies. This and the availability of health screenings across Singapore also increase the chances of the intervention being appropriate for scaling up to benefit larger segments of the population [47, 48]. Some limitations are also associated with the trial. Due to the study being embedded in ongoing community health screenings we were not able to collect accelerometer data of the primary outcome at baseline. We addressed this by controlling for self-report total PA in the secondary analysis of the primary outcome, but some small risk of unmeasured confounding remains. Blinding of participants or outcome assessors was also not possible, which we tried to address by using objective and validated measures for outcome assessments wherever possible. It is also conceivable that some contamination effects may have occurred by participants allocated to PPI group sharing information or materials with control group participants. While we tried to minimize this risk by excluding family members of existing participants from enrolment and by holding ‘closed group’ exercise sessions in parks, a minimal risk remains. However, contamination would likely result in conservative estimates of the intervention effectiveness. Furthermore, this trial was conducted in the context of community health screenings in the northern part of Singapore, which are available only to middle-aged Singaporeans and permanent residents residing in this area. Our study population is therefore not representative of the general adult population in Singapore. However, it seemed to be similar to participants of a previous survey at community health screenings [32]. Moreover, while the study was conducted in the north of the country, Singapore has a large number of parks and green spaces that are easy to access and well distributed across the island [30, 31]. We also compared key characteristics of our study population with information reported in recent national census data [49]. With regards to education level, the proportion of participants working and average household income, our study population appeared broadly similar to the adult population in Singapore. While the proportion of Chinese in our study was slightly greater and that of Malay smaller, the proportion of participants with Indian ethnicity was also similar to that in the general population. There was also no follow-up beyond 6 months, therefore longer term effects of the intervention are unknown. Finally, the distribution of certain outcome variables was skewed. Thus, we further implemented natural log-transformation on these variables to normalize data. However, the results were largely consistent with the pre-specified analyses, with conclusions remaining unchanged.

## Conclusions

Park Prescription is a novel approach to promoting time spent in nature and participation in PA for better wellbeing and health. The Park Prescription Trial appears to be the first RCT that has investigated the effectiveness of a park prescription intervention in a community-dwelling population, with some positive yet somewhat inconsistent outcomes. PPI was able to achieve substantial increases in park use, PA in parks and recreational PA, but did not achieve significant increases in the primary outcome objectively measured bouted MVPA. In this healthy, middle-aged population the trial furthermore provides evidence for possible beneficial effects of the intervention on QoL but not for psychological distress and cardio-metabolic outcomes. Prospective longitudinal studies with adequate sample size are warranted to confirm the demonstrated findings in relation to the health effects of PA in urban green spaces.

## Data Availability

Data and materials are available upon request and following approval by the National Parks Board of Singapore.
